# Developmental thyroid hormone action on pro-opiomelanocortin-expressing cells programs hypothalamic BMPR1A depletion and brown fat activation

**DOI:** 10.1093/jmcb/mjac078

**Published:** 2022-12-29

**Authors:** Zhaofei Wu, M Elena Martinez, Victoria DeMambro, Marie Francois, Arturo Hernandez

**Affiliations:** MaineHealth Institute for Research, Center for Molecular Medicine, MaineHealth, Scarborough, ME 04074, USA; MaineHealth Institute for Research, Center for Molecular Medicine, MaineHealth, Scarborough, ME 04074, USA; MaineHealth Institute for Research, Center for Molecular Medicine, MaineHealth, Scarborough, ME 04074, USA; Graduate School of Biomedical Science and Engineering, University of Maine, Orono, ME 04469, USA; Naomi Berrie Diabetes Center, Division of Molecular Genetics, Columbia University Irving Medical Center, New York, NY 10032, USA; MaineHealth Institute for Research, Center for Molecular Medicine, MaineHealth, Scarborough, ME 04074, USA; Graduate School of Biomedical Science and Engineering, University of Maine, Orono, ME 04469, USA; Department of Medicine, Tufts University School of Medicine, Boston, MA 02111, USA

**Keywords:** thyroid hormone, type 3 deiodinase (DIO3), bone morphogenetic receptor 1a (BMPR1A), pro-opiomelanocortin (POMC), hyperphagia, brown adipose tissue, corticosterone

## Abstract

Thyroid hormone excess secondary to global type 3 deiodinase (DIO3) deficiency leads to increased locomotor activity and reduced adiposity, but also to concurrent alterations in parameters of the leptin–melanocortin system that would predict obesity. To distinguish the underlying contributions to the energy balance phenotype of DIO3 deficiency, we generated mice with thyroid hormone excess targeted to pro-opiomelanocortin (POMC)-expressing cells via cell-specific DIO3 inactivation. These mice exhibit a male-specific phenotype of reduced hypothalamic *Pomc* expression, hyperphagia, and increased activity in brown adipose tissue, with adiposity and serum levels of leptin and thyroid hormones remained normal. These male mice also manifest a marked and widespread hypothalamic reduction in the expression of bone morphogenetic receptor 1a (BMPR1A), which has been shown to cause similar phenotypes when inactivated in POMC-expressing cells. Our results indicate that developmental overexposure to thyroid hormone in POMC-expressing cells programs energy balance mechanisms in a sexually dimorphic manner by suppressing adult hypothalamic BMPR1A expression.

## Introduction

Thyroid hormone (3,5,3′-triiodothyronine or T3) influences energy balance largely by regulating biochemical processes and cell physiology in metabolic tissues. Mitochondrial function (Sinha et al., 2015), glucose homeostasis (Bauer et al., 2009; Vujovic et al., 2009), and lipid and cholesterol metabolism (Astapova et al., 2014; Domouzoglou et al., 2014; Singh et al., 2017) are a few of the processes regulated by T3 in multiple tissues including the liver, muscle, and white and brown adipose tissues (WAT and BAT, respectively). In addition, T3 is critical for adequate BAT thermogenesis and *Ucp1* expression (Christoffolete et al., 2004; Ribeiro et al., 2010) and has also been shown to induce beiging in WAT (Lin et al., 2015). However, T3 also contributes to central regulation of energy balance by affecting hypothalamic regulatory circuits (Zhang et al., 2017). For instance, the administration of T3 directly into the hypothalamus causes local changes in adenosine monophosphate-activated protein kinase and fatty acid metabolism, altering paradigms of energy homeostasis (López et al., 2010). Chronic administration of T3 into the paraventricular hypothalamic nucleus leads to more limited effects depending on the duration of treatment (Zhang et al., 2016).

However, in normal rodent and human physiology, T3 availability and action in the brain is tightly controlled by the action of the type 2 and 3 deiodinase enzymes (DIO2 and DIO3, respectively) (Alkemade et al., 2008; Friesema et al., 2012). While DIO2 in astrocytes (Bocco et al., 2016) and hypothalamic tanycytes (Mihaly et al., 2000; Kwakkel et al., 2014) can convert the most abundant hormone thyroxine (T4) into the most active hormone T3, neuronal DIO3 (Tu et al., 1999) can transform both T4 and T3 into biologically inactive compounds (Hernandez, 2005). The paracrine dynamic interplay between DIO2 and DIO3 provides the brain with a mechanism to maintain T3 action within a narrow range, as appropriate to the brain region and developmental stage (Flamant et al., 2015; Hernandez et al., 2021). Additional evidence for a role of local T3 in the central regulation of energy balance comes from mice with deiodinase deficiency. Studies in *Dio2*-null mice reveal a role for local T3 generation in the activation of orexigenic neurons upon fasting, a mechanism that involves UCP2 activation (Coppola et al., 2007).

Further evidence supporting a contribution of endogenous hypothalamic T3 in the regulation of energy balance is provided by observations in mice lacking a functional DIO3. Due to impaired clearance of thyroid hormones, *Dio3*^–/–^ mice exhibit increased T3 action in the hypothalamus, abnormal energy balance (Wu et al., 2017), and alterations in critical elements of the leptin–melanocortin system, an essential component of the hypothalamic mechanisms regulating metabolism in peripheral tissues (Bjorbaek and Hollenberg, 2002; Seeley et al., 2004; Krashes et al., 2016). *Dio3*^–/–^ mice manifest increased food intake and elevated and reduced, respectively, hypothalamic expression of *Agrp* and *pro-opiomelanocortin* (*Pomc*), suggesting a positive energy balance. Intriguingly, these mice also exhibit reduced adiposity, an outcome that may be partially explained by their increased level of physical activity, possibly due to elevated T3 action in other regions of the brain (Wu et al., 2017). Moreover, *Dio3*^–/–^ mice are overexposed to T3 during fetal and neonatal life (Hernandez et al., 2006; Martinez and Hernandez, 2021), raising the possibility that a large proportion of the abnormalities have a developmental origin. We have shown that this is the case for the neurobehavioral phenotype using a model of adult-onset DIO3 deficiency (Stohn et al., 2019).

Thus, the impact of global DIO3 deficiency and subsequent T3 excess on energy balance is complex and may affect multiple systems. In order to dissect individual mechanisms by which a developmental excess of T3 affects energy balance endpoints in the adult hypothalamus, we targeted DIO3 inactivation to POMC-expressing cells using mice carrying a floxed *Dio3* allele (Martinez et al., 2019; Stohn et al., 2019) and a transgene driving the expression of cre DNA recombinase from the Pomc promoter (*Pomc-cre/Dio3^f/f^*mice). Our results reveal a sexually dimorphic phenotype, with *Pomc-cre/Dio3^f/f^*male mice exhibiting decreased bone morphogenetic protein receptor 1A (BMPR1A) in most hypothalamic areas in association with hyperphagia, decreased hypothalamic expression of *Pomc* and *Ucp2*, and BAT activation. Our results indicate that elevated T3 action in POMC-expressing cells during development has long-term and sexually dimorphic effects on the programming of hypothalamic BMPR1A and energy balance.

## Results

### DIO3 deficiency in POMC cells affects the adrenal axis, but not the thyroid axis, in males

**Figure 1:**
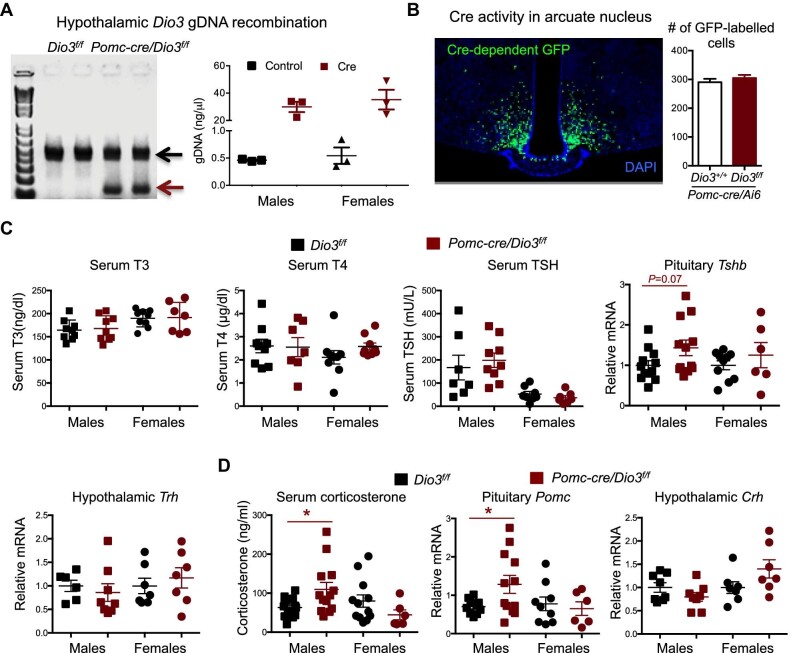
*Dio3* DNA recombination and hormonal profiles in *Pomc-cre/Dio3^f/f^*mice. (**A**) PCR and qPCR results showing the presence (left) and the abundance (right) of the recombinant *Dio3* allele. (**B**) GFP-labelled cells showing cre-mediated recombination in the hypothalamic arcuate nucleus (*n* = 3). (**C**) Serum and pituitary endpoints of the hypothalamic–pituitary–thyroid axis. (**D**) Serum and pituitary endpoints of the hypothalamic–pituitary–adrenal axis. *P < 0.05 as determined by unpaired Student's *t*-test.

To target DIO3 deficiency to POMC-expressing cells, we crossed mice expressing *cre* from the *Pomc* promoter (*Pomc-cre* mice) (Balthasar et al., 2004) with mice carrying conditional (flanked by loxP sites) *Dio3* alleles (*Dio3^f/f^* mice), as previously reported (Martinez et al., 2019; Stohn et al., 2019). Polymerase chain reaction (PCR) of genomic DNA extracted from the hypothalamus of *Pomc-cre/Dio3^f/f^* mice exhibited a distinctive PCR band specific for cre-recombined *Dio3*. This band was not present in *Dio3^f/f^* mice (Figure 1A, left). Quantification of *Dio3* gene recombination (the PCR band specific for *Dio3* recombination) using quantitative real-time PCR (qPCR) of the same hypothalamic genomic DNA showed an ∼60-fold increase in *Pomc-cre/Dio3^f/f^* mice (Figure 1A, right). Since this recombination leads to DIO3 inactivation (Martinez et al., 2019; Stohn et al., 2019), these data indicate that DIO3 is inactivated in the hypothalamus. The anatomic distribution of this inactivation was confirmed in adult mice (5 months of age) that carried a cre-dependent GFP transgene (Ai6). The latter mice showed GFP expression, i.e. DNA recombination, in distinct cells in the hypothalamic arcuate nucleus (Figure 1B), consistent with the known location of POMC-expressing cells in this region and previous reports using this cre transgenic line. Taken together, these data demonstrate that DIO3 is inactivated in cells expressing *Pomc*. There was no difference in the number of hypothalamic cells expressing the GFP transgene between control mice and mice with POMC cell-specific DIO3 deficiency (Figure 1B). We examined the thyroid and adrenal hormonal axes in these mice, given that POMC neurons mediate the action of leptin to regulate thyrotropin-releasing hormone (TRH) (Harris et al., 2001) and that adreno-corticotropin hormone (ACTH) is produced by processing of the POMC peptide. *Pomc-cre/Dio3^f/f^* mice did not exhibit abnormalities in the serum levels of thyroid hormones or thyrotropin (TSH), nor in pituitary *Tshb* or hypothalamic *Trh* mRNA expression (Figure 1C). In contrast, serum levels of corticosterone and pituitary expression of *Pomc* were significantly elevated in *Pomc-cre/Dio3^f/f^* male mice, but not in females (Figure 1D). No significant difference in hypothalamic expression of corticotropin-releasing hormone (*Crh*) mRNA was observed in mutant mice of either sex (Figure 1D).

### Alterations in hypothalamic regulators of energy balance

**Figure 2:**
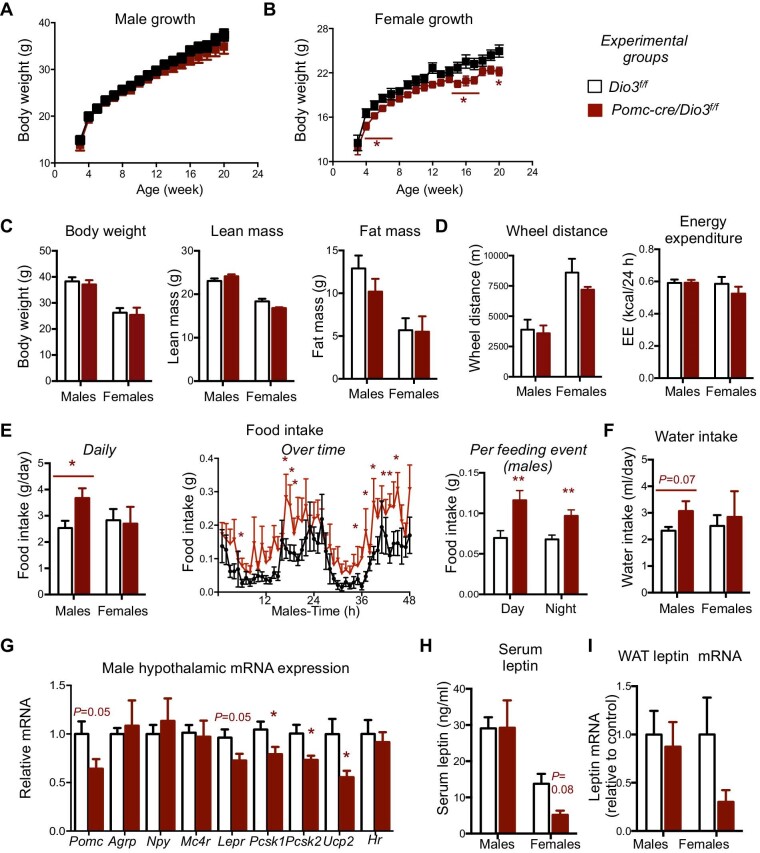
Growth, metabolism, and hypothalamic markers in *Pomc-cre/Dio3^f/f^* mice. (**A**) Growth of male mice. (**B**) Growth of female mice. (**C**) Weight and body composition of *Pomc-cre/Dio3^f/f^* and control mice. (**D**) Physical activity and energy expenditure. (**E**) Food intake in *Pomc-cre/Dio3^f/f^* and control mice. (**F**) Water intake in *Pomc-cre/Dio3^f/f^* and control mice. (**G**) Hypothalamic gene expression in male mice. (**H**) Serum leptin. (**I**) Leptin mRNA expression in WAT. *P < 0.05 as determined by unpaired Student's *t*-test (*n* = 7–14).

After weaning and up to 22 weeks of age, the growth of *Pomc-cre/Dio3^f/f^* male mice was not significantly different from that of controls, although body weight trended lower with age (Figure 2A). *Pomc-cre/Dio3^f/f^* female mice showed slightly reduced growth, although a reduction in body weight was statistically significant only for 3 weeks after weaning and at later ages (Figure 2B). At 18 weeks of age, we observed no differences in body weight, lean mass, or fat mass (Figure 2C). We then subjected these animals to studies in metabolic cages. The results revealed no significant differences in physical activity, total energy expenditure (Figure 2D; Supplementary Table S1), or rectal temperature (36.8 ± 0.442 vs. 36.8 ± 0.319 in control male mice, *n* = 7 and 8, respectively) in experimental mice of either sex. *Pomc-cre/Dio3^f/f^* males still showed decreased energy expenditure during the day time period of lower activity (Supplementary Figure S1A). However, we observed a sexually dimorphic effect on substrate fuel utilization where the night-time and 24-h average resting respiratory exchange ratios (RER) were closer to 0.8 in *Pomc-cre/Dio3^f/f^* male mice, indicative of oxidation of a mix of protein, carbohydrates, and fat. In females, RER values were closer to 0.7 (*P* = 0.06–0.1), suggesting fatty acid oxidation as the primary fuel source (Supplementary Figure S1B). Food intake was significantly elevated in *Pomc-cre/Dio3^f/f^* males, but not in females (Figure 2E). This increase in food intake was observed during both the light and dark cycles, consistent with increased food intake per feeding event (Figure 2E). The hyperphagia in mutant males was associated with elevated water intake, although the latter did not reach statistical significance (*P* = 0.07) (Figure 2F). We also found that hypothalamic parameters of relevance to energy balance were altered in males. *Pomc-cre/Dio3^f/f^* males exhibited reductions in the expression of *Pomc, Lepr, Ucp2*, and peptide convertase genes *Pcsk1* and *Pcsk2* (Figure 2G), while immunofluorescence (IF) of POMC fiber projections did not exhibit any notable abnormality (data not shown). Hypothalamic epression of *Agrp, Npy*, and *Mc4r* was unchanged (Figure 2G). Expression of the T3-dependent gene *Hr* was not altered, suggesting no changes in the average levels of T3 signaling across the hypothalamus. To further confirm this point, we measured the hypothalamic expression of additional eight genes that are among the top genes more consistently regulated by thyroid hormone in brain tissue, according to a recent compendium (Chatonnet et al., 2015). We did not observe significant changes in the expression of any of them (Supplementary Figure S1C). Also, no significant changes in gene expression related to energy balance were observed in the hypothalamus of females (Supplementary Figure S1D). The alterations in male hypothalamic markers occurred in the absence of significant changes in serum leptin (Figure 2H) or leptin mRNA expression levels in WAT (Figure 2I). However, both parameters trended lower in *Pomc-cre/Dio3^f/f^* females.

### Decreased hypothalamic BMPR1A expression in *Pomc-cre/Dio3^f/f^* male mice

**Figure 3:**
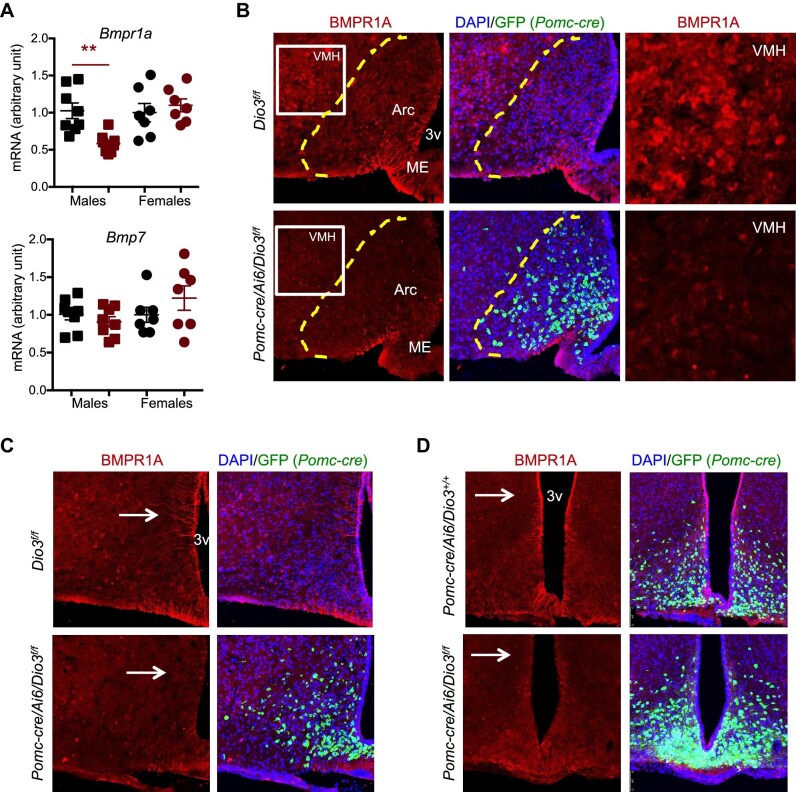
Hypothalamic expression of BMPR1A in 5-month-old *Pomc-cre/Dio3^f/f^* mice. (**A**) *Bmpr1a* mRNA expression in the overall hypothalamus. (**B**) BMPR1A IF in the median eminence, arcuate nucleus, and VMH of male mice. (**C**) BMPR1A IF in the VMH and tanycytes of male mice. (**D**) BMPR1A IF in the hypothalamic tanycytes lining the third ventricle of male mice. **P < 0.01 as determined by unpaired Student's *t*-test. Images are representative of 2 or 3 mice per genotype. Arc, arcuate nucleus; ME, median eminence; 3v, third ventricle.

**Figure 4:**
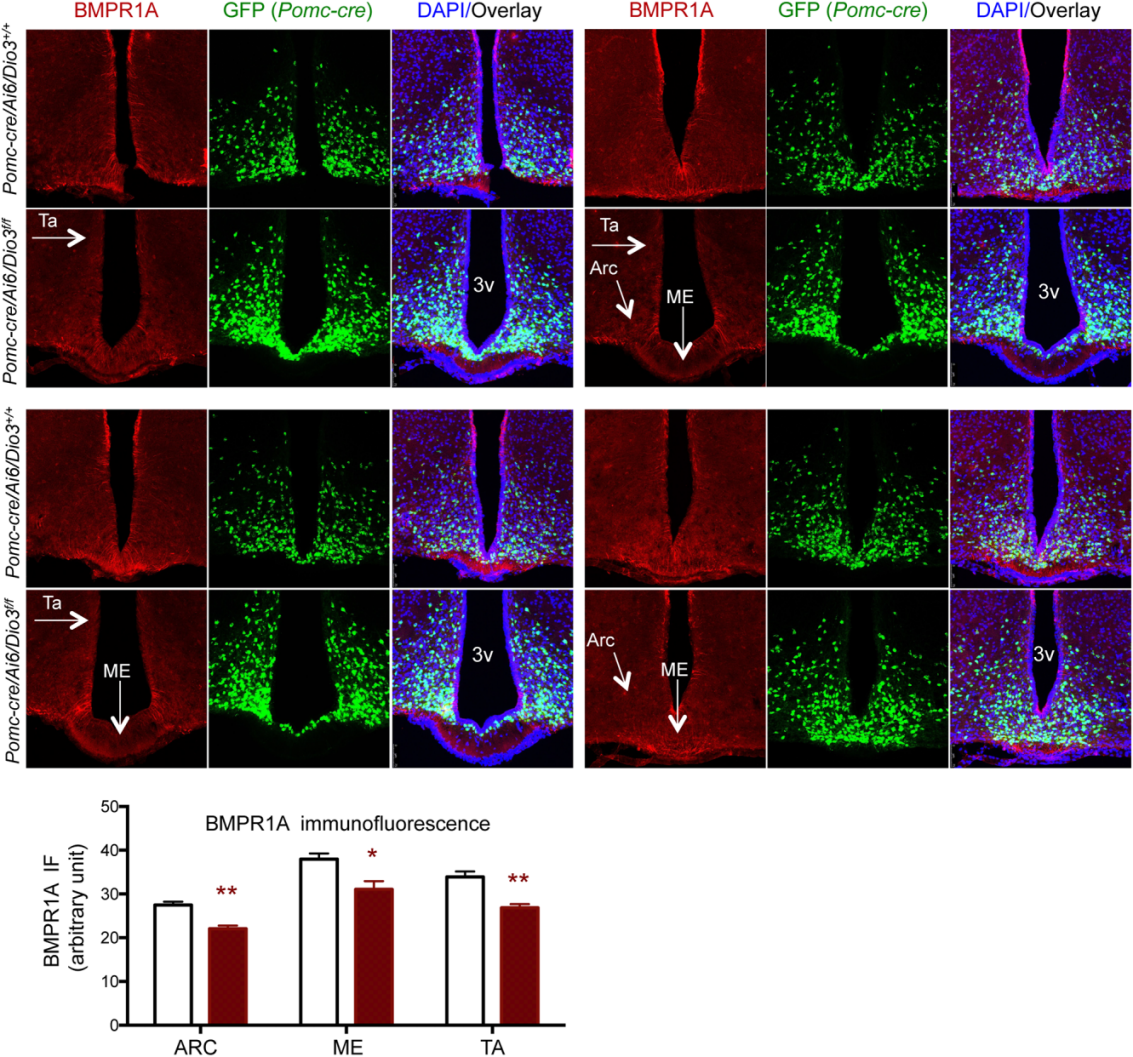
BMPR1A IF in 3-week-old mice. Decreased BMPR1A IF is observed in several hypothalamic regions (arrows), including the arcuate nucleus, the median eminence, and tanycytes lining the third ventricle. First and third rows: control mice; second and fourth rows: mice with POMC neuron-specific DIO3 deficiency. Quantification showed no significant difference in the number of POMC neurons between experimental groups (not shown). Quantification of BMPR1A IF was performed in three sections of four different mice. *P < 0.05 and **P < 0.01 as determined by the Student's *t*-test (*n* = 4). Arc, arcuate nucleus; ME, median eminence; Ta, tanycytes; 3v, third ventricle.

**Figure 5:**
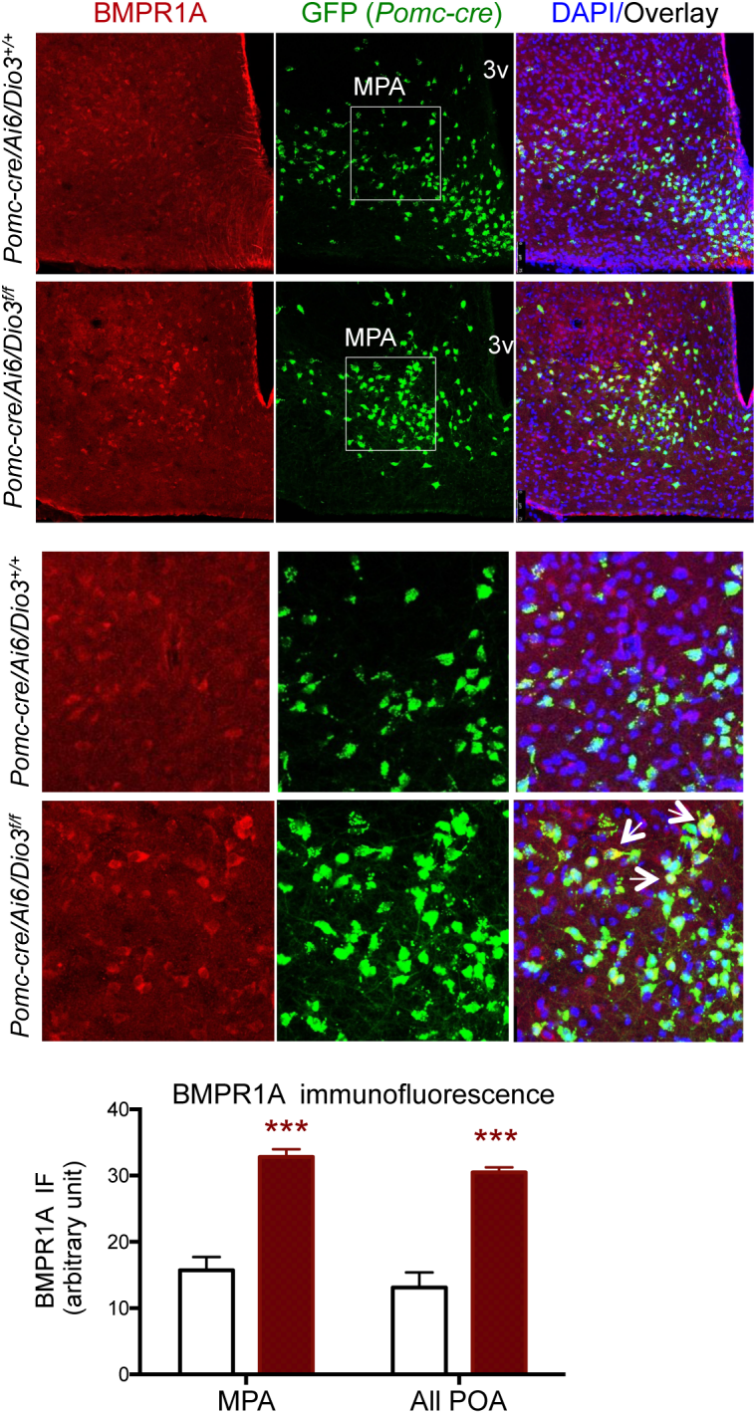
Hypothalamic BMPR1A IF in 3-week-old mice. The medial preoptic area of *Pomc-cre/Ai6/Dio3^f/f^* mice (bottom row) exhibits elevated BMPR1A IF and increased number of POMC-expressing cells compared to that of control mice (top row). Substantial co-localization of BMPR1A and POMC is also observed in many cells within this hypothalamic region. Quantification of BMPR1A IF was performed in two sections of three and five different mice, respectively. ***P < 0.001 as determined by the Student's *t*-test (*n* = 3 and 5). MPA, medial preoptic area; 3v, third ventricle.

An important hypothalamic determinant of energy balance is BMPR1A, which has been shown to play a role in the development of hypothalamic circuits affecting food intake (Peng et al., 2012). Of particular relevance to our present studies, BMPR1A has been shown to regulate food intake and enhance the activity of BAT when it is depleted specifically in POMC-expressing cells (Townsend et al., 2017). In this regard, qPCR experiments showed a significant decrease in *Bmpr1a* mRNA expression in the hypothalamus of adult (5 months old) *Pomc-cre/Dio3^f/f^* male mice but not in females, while the expression of *Bmp7*, the BMPR1A endogenous ligand, was not affected in either sex (Figure 3A). Hypothalamic BMPR1A IF largely co-localized to POMC-expressing cells and revealed marked reductions in BMPR1A in the arcuate nucleus of *Pomc-cre/Dio3^f/f^* male mice (Figure 3B). Interestingly, reductions in BMPR1A IF extended to other hypothalamic areas where POMC neurons are not normally present, including the ventromedial hypothalamus (VMH) (Figure 3B), the median eminence, and tanycytes lining the dorsal region of the third ventricle (Figure 3C and D). Reduced BMPR1A IF in median eminence, arcuate nucleus, and tanycytes was already observed at weaning age in *Pomc-cre/Dio3^f/f^* male mice (Figure 4), suggesting a developmental origin. Intriguingly, we also observed at this age increased number of POMC-expressing cells and elevated BMPR1A IF in the medial preoptic area of *Pomc-cre/Dio3^f/f^* male mice (Figure 5), suggesting mislocalization of some POMC-expressing cells. BMPR1A IF co-localized to POMC-expressing cells (Figure 5).

**Figure 6:**
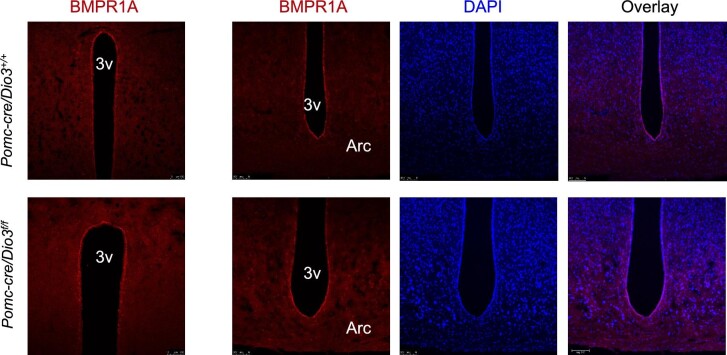
BMPR1A IF in 10-month-old female mice. As in younger adult females, no appreciable difference in BMPR1A IF is observed in tanycytes lining the third ventricle or in the arcuate nucleus. Arc, arcuate nucleus; 3v, third ventricle.

**Figure 7:**
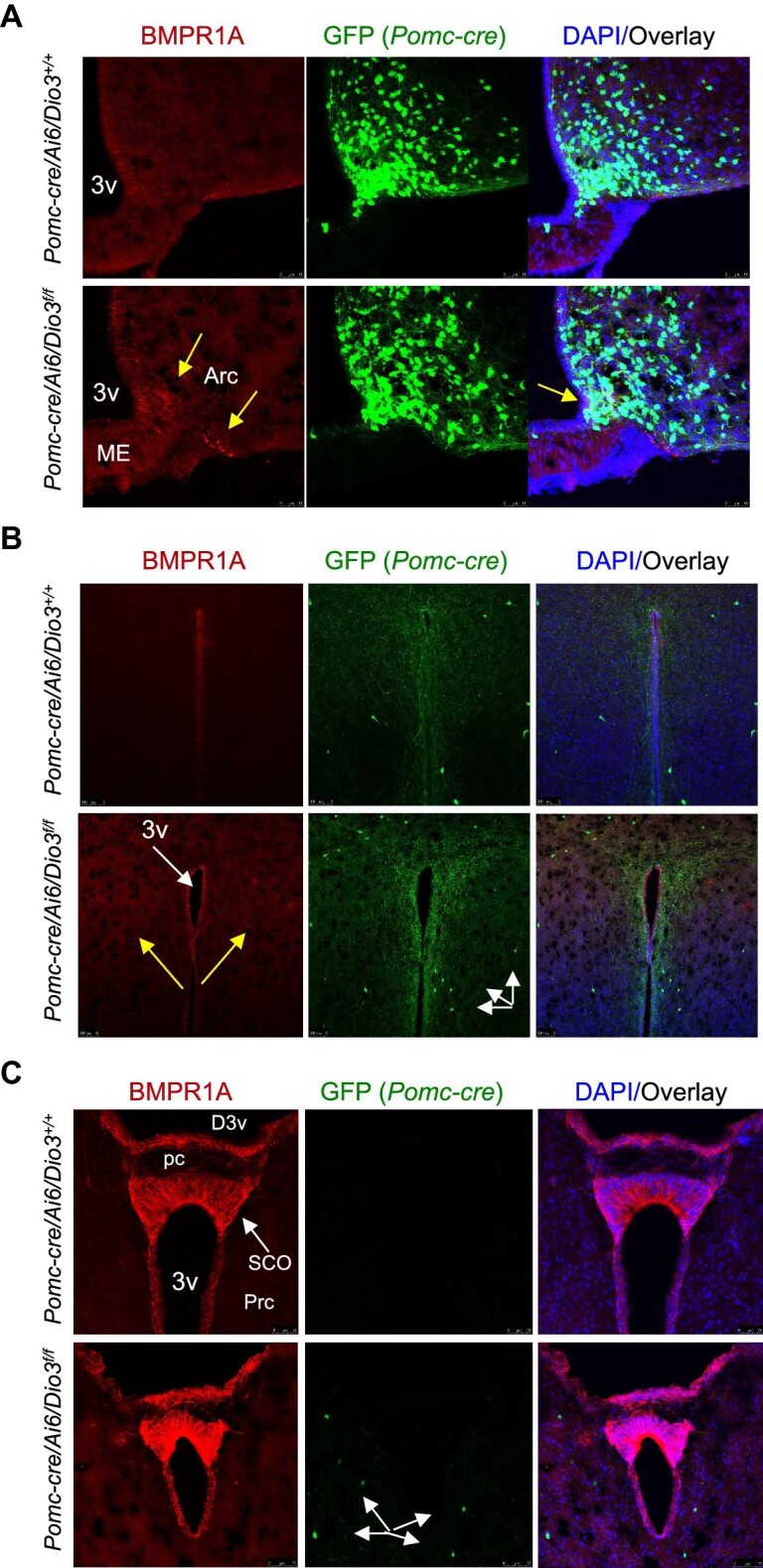
BMPR1A IF in 15-day-old female mice. (**A**) Increased BMPR1A IF and BMPR1A cell number (yellow arrows, left) in *Pomc-cre/Ai6/Dio3^f/f^* female mice compared to controls, with partial co-localization with POMC-expressing cells (yellow arrow, right). (**B**) Increased BMPR1A IF (yellow arrows, left) in the paraventricular region. POMC-expressing cells are indicated by white arrows (center). (**C**) Strong BMPR1A IF in the subcommissural organ and in cells lining the third and dorsal third ventricles, with *Pomc-cre/Ai6/Dio3^f/f^* female mice showing a few POMC-expressing cells in the precommissural nucleus area (white arrows, center), while none is observed in control mice. Arc, arcuate nucleus; ME, median eminence; 3v, third ventricle; D3v, dorsal third ventricle; pc, posterior commissure; SCO, subcommissural organ; Prc, precommissural nucleus.

In *Pomc-cre/Dio3^f/f^* females, we did not observe changes in hypothalamic BMPR1A IF at 10 months of age (Figure 6). However, in contrast to what was observed in male weanlings (Figure 4), 15-day-old *Pomc-cre/Dio3^f/f^* females exhibited increased BMPR1A expression in cells in the arcuate nucleus and median eminence (Figure 7A) as well as in tanycytes (Figure 7B). We detected increased BMPR1A IF in the paraventricular region (Figure 7B). In addition, strong BMPR1A expression was noted at this age in the subcommissural organ and the expression also appeared higher in *Pomc-cre/Dio3^f/f^* females (Figure 7B). No apparent differences in BMPR1A IF between genotypes were noted in cells lining the dorsal third ventricle. Interestingly, some POMC-expressing cells were observed in the region of the precommissural nucleus (Figure 7C) only in *Pomc-cre/Dio3^f/f^* females but not in controls, suggesting a mislocalization of POMC-expressing cells.

### Activated BAT in *Pomc-cre/Dio3^f/f^* male mice

**Figure 8:**
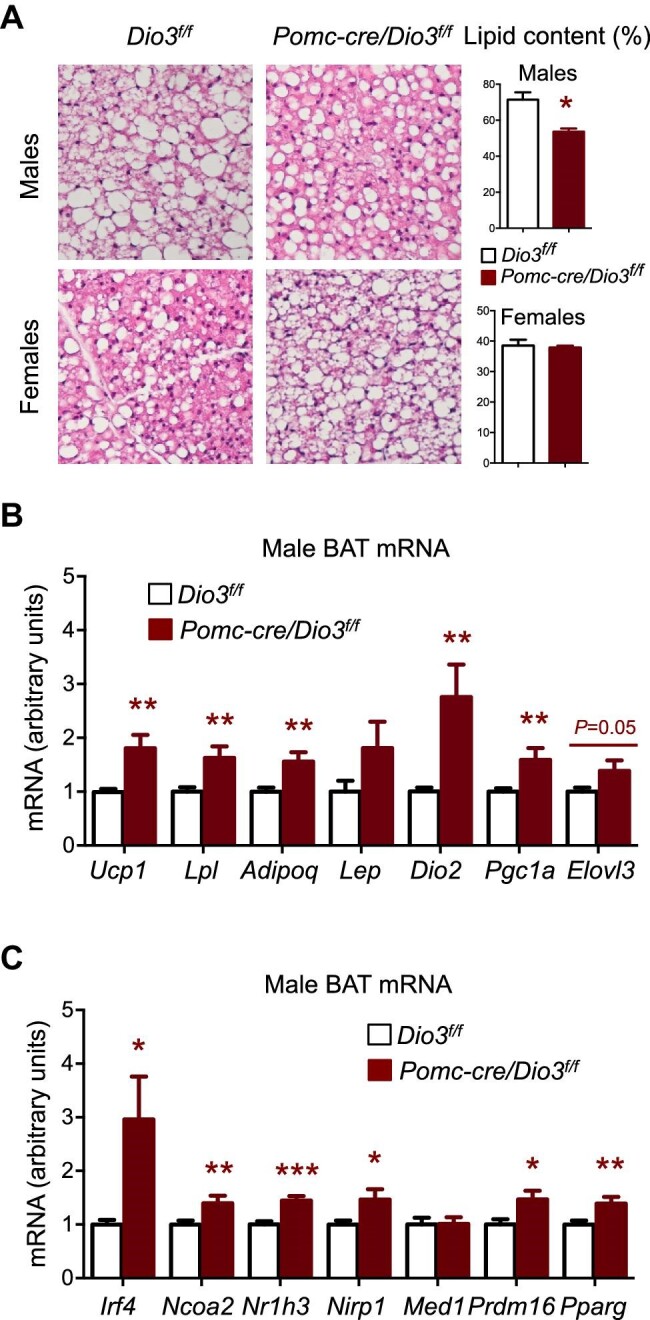
BAT activation in *Pomc-cre/Dio3^f/f^* male mice. (**A**) H&E staining and lipid content of BAT from male and female *Pomc-cre/Dio3^f/f^* mice compared to controls. (**B**) Expression of genes related to BAT activation in male mice. (**C**) Expression of genes related to BAT recruitment in male mice. *P < 0.05, **P < 0.01, and ***P < 0.001 as determined by unpaired Student's *t*-test. Images are representative of 2 or 3 mice per genotype.

Consistent with the male-specific decreased hypothalamic BMPR1A expression, an activation of BAT in *Pomc-cre/Dio3^f/f^* male mice, but not in females, was observed. Hematoxylin and eosin (H&E) staining of *Pomc-cre/Dio3^f/f^* BAT showed smaller brown adipocytes and reduced lipid content in male mice, but not in females (Figure 8A), suggesting BAT activation in male mice with POMC cell-specific DIO3 deficiency. This was confirmed by BAT data showing elevated expression of genes related to adrenergic stimulation and thermogenesis, including *Ucp1, Lpl, Dio2, Pgc1a, Adipoq*, and *Elovl3* (Figure 8B). The BAT activation signature of *Pomc-cre/Dio3^f/f^* males was associated with increased expression of genes related to BAT recruitment, including *Irf4, Ncoa2, Nr1h3, Nirp1, Prdm16*, and *Pparg* (Figure 8C).

## Discussion

The important role of thyroid hormone in regulating cell metabolism in most organs has long been known, but their function in the central regulation of energy balance has also been increasingly appreciated (Coppola et al., 2007; López et al., 2010). The observation in rodents that exogenous T3 administration into the hypothalamus impacts local and peripheral metabolism (López et al., 2010) raised the possibility that endogenous factors regulating T3 availability in this brain region may also influence metabolic phenotypes. We previously observed that lacking one of these factors, DIO3, results in decreased adiposity and abnormalities in serum leptin and hypothalamic expression of *Pomc* and other genes of metabolic relevance (Wu et al., 2017). However, global DIO3 deficiency causes a complex developmental syndrome affecting multiple endocrine systems (Hernandez et al., 2007; Medina et al., 2011) and physical activity (Wu et al., 2017), confounding the distinct effects of T3 excess during early life on developing hypothalamic circuitries that will regulate energy balance in adulthood.

Here, we used a genetic approach of conditional DIO3 inactivation to achieve T3 excess specifically in POMC-expressing cells. Mature POMC neurons are key to rely the anorexigenic effects of leptin to other hypothalamic circuitries (Ahima and Osei, 2004). These effects include the activation of the thyroid axis through induction of TRH in the paraventricular nucleus (Harris et al., 2001; Guo et al., 2004). However, we observed that *Pomc-cre/Dio3^f/f^* mice do not exhibit any alterations in thyroid hormone status at least at baseline, suggesting that metabolic phenotypes in *Pomc-cre/Dio3^f/f^* mice are not resulting from abnormal thyroid hormone action in peripheral tissues. Metabolic studies in these mice indicate no major alterations in growth, adiposity, or energy expenditure. Still, the thyroid axis and adiposity outcomes of these animals may be abnormal in response to specific physiological challenges, and this susceptibility will need to be investigated. Consistent with the normal levels of circulating thyroid hormones, T3 action in the hypothalamus is also unaltered in *Pomc-cre/Dio3^f/f^* mice, as determined by average expression levels of T3 target genes. This is not surprising considering that most hypothalamic cells are targets of thyroid hormone, but POMC neurons, where T3 clearance is impaired, represent a very small proportion of total hypothalamic cells.

However, similar to that described in *Dio3*^–/–^ mice (Wu et al., 2017), *Pomc-cre/Dio3^f/f^* male mice are hyperphagic and manifest abnormalities in hypothalamic gene expression. *Pomc* mRNA expression is decreased, further supporting this phenotype as a distinct outcome of hypothalamic T3 excess during development. Hypothalamic expression of *Ucp2* and peptide convertase genes *Pcsk1* and *Pcsk2* are also decreased. In the face of increased food intake, we do not note elevated expression of *Npy* or *Agrp*, although it is possible that the activity of AGRP neurons is increased. Considering that null mice for *Pcsk1* and *Pcsk2* do not exhibit adiposity phenotypes (Shakya et al., 2021), it is unlikely that the observed reductions in their expression contribute to an energy balance phenotype. On the other hand, reduced expression of *Pomc* (Bjorbaek and Hollenberg, 2002) and *Ucp2* (Coppola et al., 2007) as well as hyperphagia would predict, together with the slight decrease in leptin receptor expression, a positive energy balance and obesity, possibly due to partial leptin resistance. However, although the latter possibility needs to be directly studied, no obesity phenotype is observed, suggesting a compensatory mechanism that promotes energy expenditure.

Since there is no significant difference in physical activity between control and *Pomc-cre/Dio3^f/f^* mice, decreased hypothalamic expression of BMPR1A may be a part of such compensatory mechanism. Townsend et al. (2017) recently described that BMPR1A deficiency specifically in POMC neurons leads to the increased adrenergic tone and BAT activation. Likewise, we also observed a marked decrease in BMPR1A expression in the hypothalamus of *Pomc-cre/Dio3^f/f^* male mice, and this deficit also affects POMC neurons. Furthermore, this is associated with a robust profile of increased BAT activity based on histological and specific gene expression data, mimicking the results from the POMC cell-specific model of BMPR1A deficiency (Townsend et al., 2017). Interestingly, we observed that the BMPR1A deficiency of *Pomc-cre/Dio3^f/f^* mice not only affects POMC neurons but also extends to multiple areas of the hypothalamus including tanycytes, arcuate nucleus, and median eminence, which may influence neuroendocrine function and energy balance. Thus, in our model, other hypothalamic circuitries impacting energy balance and dependent on BMPR1A signaling may also be affected. For instance, we observed reduced BMPR1A expression in the VMH, a nucleus involved in several hypothalamic circuitries that regulate glucose homeostasis (Sejling et al., 2021; Sutton et al., 2021), food intake (Nguyen et al., 2020; Zhang et al., 2020), and leptin sensitivity (Berger et al., 2016). BMPR1A also has a role in the development of orexigenic neurons in the hypothalamus, including their projections to the VMH (Peng et al., 2012). Moreover, a reduction in thyroid hormone signaling in the VMH by knockdown of the thyroid hormone beta receptor leads to hyperphagia, reduced energy expenditure, and obesity (Hameed et al., 2017). Thus, it is likely that the abnormal BMPR1A signaling during development leads to aberrant programming of the VMH, with subsequent consequences for multiple mechanisms of energy balance regulation. Lastly, the apparent ectopic localization of some POMC cells in the medial preoptic area and the subcommissural organ raises the possibility that DIO3 deficiency affects the migration and ultimate anatomic location of a subset of these cells, with unknown functional implications.

Although energy expenditure is higher in *Pomc-cre/Dio3^f/f^* male mice than in controls, the difference is not statistically significant, as one would predict as a result of hyperphagia and BAT activation. It is possible that alterations in other central mechanisms have compensatory effects on energy expenditure that need to be identified. For example, alterations in leptin signaling in vagal afferent neurons (Cakir et al., 2007; Huang et al., 2021) or in the melanocortin receptors in the vagus nerve (Liu et al., 2003) may be of consequence for the regulation of the gastro-intestinal tract (Browning and Carson, 2021; Clyburn and Browning, 2021). This raises the possibility that vagus nerve alterations may diminish nutrient absorption (Mourad and Saadé, 2011; Huang et al., 2021). This diminished effective calorie intake may lead to reduced energy expenditure in other tissues that would compensate for BAT activation.

The alterations in *Pomc-cre/Dio3^f/f^* mice discussed above are sexually dimorphic and affect males, but not females. These alterations appear to be present already at weaning age, indicating that the result of malprogramming occurs earlier in life, possibly affecting neonatal hypothalamic sexual differentiation (Tobet, 2002). Most sexual dimorphisms in the brain, including the hypothalamus, arise as a result of the neonatal peak in testosterone and subsequent action of this hormone in the male brain, largely after it is locally converted to estradiol (Balthazart and Ball, 1998; Schwarz and McCarthy, 2008). Considering the marked neonatal peak of *Dio3* expression in the rodent hypothalamus (Kaplan and Yaskoski, 1981; Hernandez et al., 2006), it is possible that sexually dimorphic processes involving POMC-expressing cells are affected by loss of DIO3 function and the subsequent increase in T3 action, as T3 has been reported to cross-talk with hypothalamic estradiol signaling (Dellovade et al., 1999, 2000; Vasudevan et al., 2001, 2002; Faustino et al., 2015).

It is also important to note that cells that express POMC during development not only give rise to adult POMC neurons but also to other types of hypothalamic neurons (Padilla et al., 2010), raising the possibility that thyroid hormone excess in POMC cells early in development leads to abnormalities in the adult hypothalamus not restricted to POMC neurons. This is also consistent with our observations here suggesting anomalies in the anatomic localization of some POMC cells in *Pomc-cre/Dio3^f/f^* mice.

Although *Dio3* is expressed in neural progenitors and mature neurons (Escamez et al., 1999; Tu et al., 1999), it is also expressed in other tissues, especially during development (Hernandez et al., 2021). Likewise, *Pomc* is also expressed in other mouse tissues. It is unknown whether significant *Dio3* expression is present in other types of POMC-expressing cells, but this possible limitation needs to be considered. DIO3 loss of function in non-hypothalamic tissues may influence the phenotype of the mouse model studied here. In this regard, the case of the pituitary is particularly relevant. Pituitary processing of POMC generates ACTH, a pituitary hormone critical for the regulation of corticosteroid production in the adrenal gland. It is not known whether *Dio3* is expressed in ACTH-producing pituitary cells but, if this were the case, thyroid hormone excess in ACTH-producing cells of *Pomc-cre/Dio3^f/f^* pituitaries may lead to abnormalities in the adrenal axis. This is a likely possibility considering our observation of a sexually dimorphic phenotype in adult serum corticosterone, with males exhibiting a significant increase over controls, while female values trending lower than controls. It is also possible that altered levels of corticosterone affect the metabolism of peripheral tissues and overall energy balance.

In summary, we show that lack of T3 clearance in developing POMC-expressing cells leads to sex-specific alterations in the programming of multiple hypothalamic systems. Some of these alterations remain to be elucidated, but here we report BMPR1A deficiency in most hypothalamic regions and reduced *Pomc* expression that manifest as BAT activation and hyperphagia. Considering the relatively high prevalence of thyroid disease in women of reproductive age, our results shed some light on how POMC cells might be affected by developmental thyrotoxicosis, with long-term consequences for energy balance regulation and susceptibility or resistance to obesity.

## Materials and methods

### Mouse models

Experimental *Dio3^f/f^* mice used in these studies were generated and genotyped as previously described (Hernandez et al., 2006). *Dio3^f/f^* mice on a C57Bl/6J genetic background were crossed with mice carrying a transgene expressing cre DNA recombinase under the control of the *Pomc* promoter (Stock no: 010714, Jackson Lab) to obtain *Pomc-cre/Dio3^f/+^* mice. Since original *Pomc-cre* mice were on an FVB genetic background, the latter animals were then crossed with *Dio3^f/f^* mates for three generations before generating experimental animals for study, *Pomc-cre/Dio3^f/f^* mice. We used *Dio3^f/f^* mouse littermates as controls. Both males and females were used, and the animals studied belonged to litters that were 7–9 in size. The main studies were performed in adult animals at ∼4.5–5 months of age. Animals were kept under a 12-h light cycle, fed ad libitum, and euthanized by asphyxiation with CO_2_. Blood was taken from the inferior vena cava, and serum was obtained by centrifugation and stored at −70°C until use. Likewise, fresh tissues were harvested, frozen on dry ice, and stored at −70°C until use. All animal procedures were approved by the Maine Medical Center Research Institute Institutional Animal Care and Use Committee.

### Serum determinations

Serum total T4 and T3 concentrations were determined using the total T4 and T3 Coat-a-Count RIA kits from Diagnostics Products Corp. according to the manufacturer's instructions. Serum corticosterone was determined using the AssayMax^TM^ ELISA Kit from Assaypro according to the manufacturer's instructions. Serum TSH was determined in the laboratory of Samuel Refetoff at the University of Chicago, as previously described (Pohlenz et al., 1999).

### H&E staining of WAT and BAT

WAT and BAT were fixed in 4% paraformaldehyde (PFA) and embedded in paraffin. Sections were visualized by H&E staining. Nuclear staining in brown fat was quantified in seven consecutive sections spanning the areas depicted from three different mice (21 sections per group in total) (Hernandez et al., 2010).

### RNA preparation and qPCR

Tissues were harvested and subsequently frozen on dry ice, and total RNA was extracted using the RNeasy kit from Qiagen. Total RNA (1 μg) was reverse-transcribed with M-MLV reverse transcriptase in the presence of random decamers (both from Thermo Fisher Scientific) at 65°C for 5 min and then 37°C for 50 min. The 20-μl reverse transcription reactions were diluted by adding 230 μl DNase and RNase-free water. An aliquot of each sample was mixed together for an internal standard and diluted 4-fold. qPCR reactions were set up in duplicate with gene-specific primers and SYBR Select Master Mix (Thermo Fisher Scientific) and run on the CFX Connect from BioRad, where they underwent an initial 10 min denaturing step, followed by 36 cycles of a denaturing step (94°C for 30 sec) and an annealing/extension step (60°C for 1 min). For each individual sample, expression was corrected by the expression of control housekeeping genes (*Gapdh, Actb*, or *Rn18s)*, which did not exhibit any significant difference in expression between genotypes. Expression data are shown in arbitrary units and represented as fold-increase over the mean value in the control group unless otherwise stated. Primers used are listed in Supplementary Table S2.

### Body composition and metabolic determinations

Fat mass and lean mass were measured in isoflurane-anesthetized mice using a Lunar PIXImus II DEXA Densitometer. For metabolic determinations, we utilized a Promethion metabolic cage system (Sable Systems). A standard 12-h light/dark cycle was maintained throughout the calorimetry studies and all animals had ad libitum access to standard rodent chow and water throughout the study. Prior to data collection, animals were acclimated to the cages for 24 h. The calorimetry system consisted of 16 metabolic cages (identical to home cages with bedding) equipped with water bottles, food hoppers, and home shelters connected to load cells for food and water intake as well as body weight monitoring throughout the study. All cages contained running wheels (4.5″ (11.5 cm) diameter, MiniMitter) wired to record revolutions/second continuously using a magnetic reed switch and standard XYZ beambreak assembly to monitor cage activity. Data were acquired with Metascreen v2.3.15.11 and analyzed using Expedata v1.9.27 (Sable Systems) as previously described (DeMambro et al., 2015). ANCOVA analysis between body composition and energy expenditure parameters was performed. No significant effects of body composition were found between energy expenditure and any of the variables. Thus, energy expenditure is reported in kcal/h.

### IF

Male mice at 3 weeks and 2 months of age were anesthetized with an intraperitoneal injection of 2.5% avertin (Sigma-Aldrich) phosphate-buffered saline (PBS) solution (250 mg/kg). Mice were then perfused with ice-cold PBS and 4% PFA through the heart left ventricle. The perfused brains were removed, fixed overnight in 4% PFA at 4°C, transferred to a 30% sucrose PBS solution, and stored at 4°C. Once the brains sank to the bottom of the vials, they were frozen, embedded in OCT, and stored at −70°C. The 20 μm sections were prepared using a Leica cryostat and BMPR1A IF was performed as previously described by Townsend et al. (2017). IF signal in confocal images was analyzed with NIH-developed open software package ImageJ V1.53q. Signals in subareas were manually selected and quantified with the software in 2–3 tissue sections from 3–5 different animals per experimental group.

### Statistics

Statistical significance between groups was determined by the two-tailed Student's *t*-test (two groups) or one-way ANOVA (multi-groups) using GraphPad Prism 6 (GraphPad Software Inc.). Regression analysis was performed using JMP 11.0 (SAS Institute Inc.). Significance was established at *P* ≤ 0.05 (two-tailed). Correlations are reported as Pearson *r* values (Kaiyala et al., 2010; Kaiyala and Schwartz, 2011).

## Supplementary Material

mjac078_Supplemental_File
