# Supplementary material for: Developmental thyroid hormone action on pro-opiomelanocortin-expressing cells programs hypothalamic BMPR1A depletion and brown fat activation
Source: J Mol Cell Biol. 2022 Dec 29;14(9):mjac078. doi: 10.1093/jmcb/mjac078 (PMC9982511; doi:10.1093/jmcb/mjac078)
Supplement: mjac078_Supplemental_File [file mjac078_supplemental_file.pdf]

Supplementary material

Developmental thyroid hormone action on pro-opiomelanocortin-expressing cells programs hypothalamic BMPR1A depletion and brown fat activation

Running title: POMC cell thyroid hormone hypothalamic programming

Zhaofei Wu[1], M. Elena Martinez[1], Victoria DeMambro[1,4], Marie Francois[2], and Arturo Hernandez[1,3,4]

[1] MaineHealth Institute for Research, MaineHealth, Scarborough, ME 04074, USA

[2] Naomi Berrie Diabetes Center, Division of Molecular Genetics, Columbia University Irving Medical Center, New York, NY 10032, USA

[3] Department of Medicine, Tufts University School of Medicine, Boston, MA 02111, USA

[4] Graduate School of Biomedical Science and Engineering, University of Maine, Orono, ME 04469, USA

* Correspondence to: Arturo Hernandez, Ph.D., Faculty Scientist III, Maine Medical Center Research Institute, Scarborough, ME 04074, USA; E-mail: hernaa@mmc.org; Tel: 1-207-396-8139; Fax: 1-207-396-8110; ORCID ID: 0000-0002-6314-3987

Supplementary Figure S1

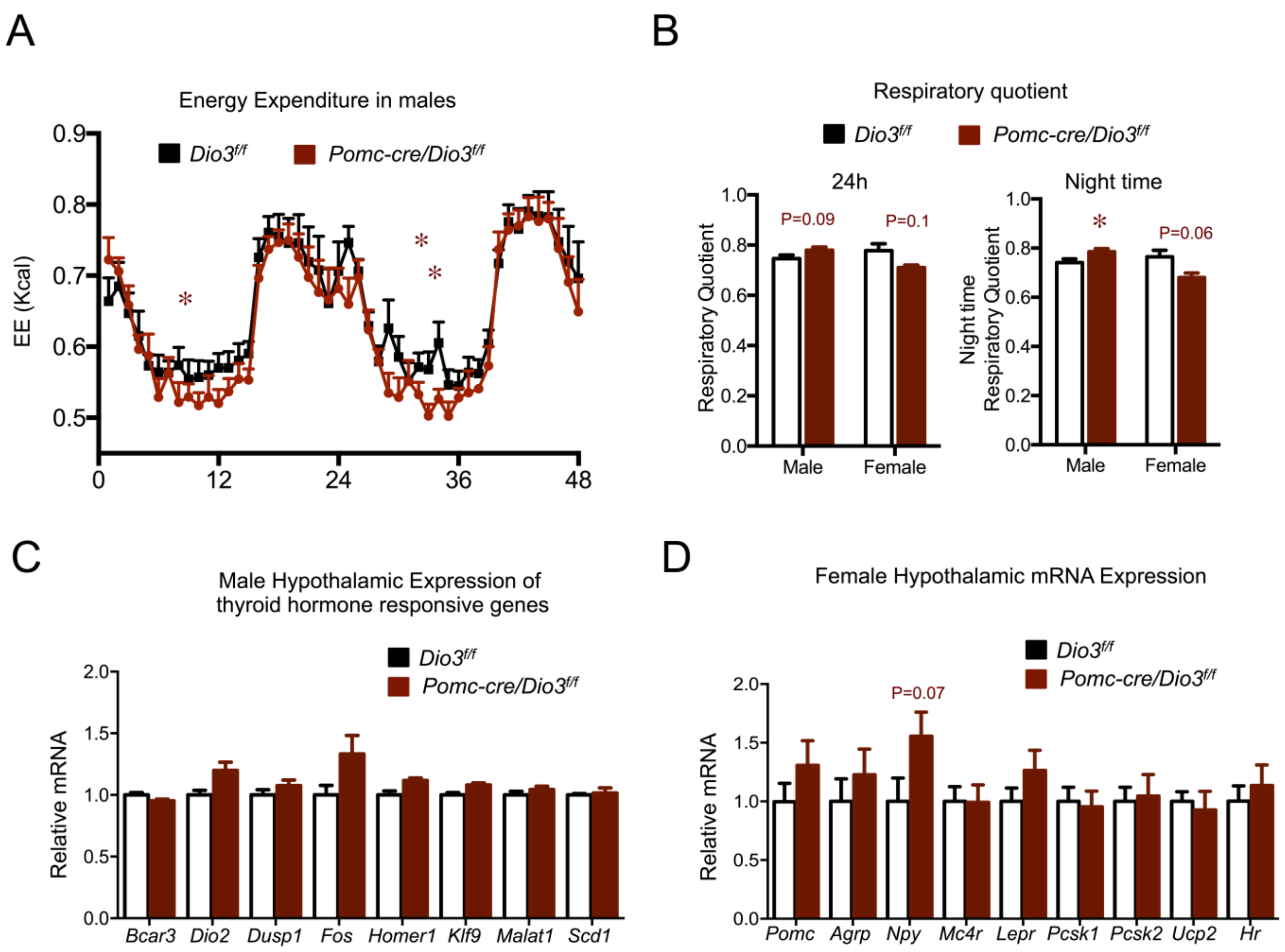

Supplementary Figure 1. (A) Circadian energy expenditure in adult males. (B) Respiratory quotients in adult mice. (C) Adult female hypothalamic gene expression of genes relevant to energy balance. (D) Adult male hypothalamic expression of top thyroid hormone responsive genes. Data represent the mean ± SEM of 7-8 males and 4 females per experimental group. *, P<0.05 as determined by the Student's t-test.

Supplementary Table S1

Abbreviation	Description	Units
**D_Avg_EE**	Energy Expenditure Daytime	**(Kcal/hr)**
**N_Avg_EE**	Energy Expenditure Night	**(Kcal/hr)**
**24_Avg_EE**	/24 hr Energy Expenditure	**(Kcal/hr)**
**D_Avg_VO2**	Daytime O2 consumed	**(ml/min)**
**N_Avg_VO2**	Night O2 consumed	**(ml/min)**
**24_Avg_VO2**	/24 hr O2 consumed	**(ml/min)**
**D_Avg_VCO2**	Daytime CO2 expelled	**(ml/min)**
**N_Avg_VCO2**	Night CO2 expelled	**(ml/min)**
**24_Avg_VCO2**	/24 hr CO2 expelled	**(ml/min)**
**D_Avg_RQ**	Daytime Respiratory Quotient	**ratio**
**N_Avg_RQ**	Nighttime Respiratory Quotient	**ratio**
**24_Avg_RQ**	/24 hr Respiratory Quotient	**ratio**
**D_QR_EE_30**	Daytime Resting Energy Expenditure (quiet Channel)	**(Kcal/30 min)**
**N_QR_EE_30**	Nighttime Resting Energy Expenditure (quiet Channel)	**(Kcal/30 min)**
**24_QR_EE_30**	/24 hr Resting Energy Expenditure (quiet Channel)	**(Kcal/30 min)**
**D_QR_RQ_30**	Daytime Resting Respiratory Quotient (quiet Channel)	**ratio/30 min**
**N_QR_RQ_30**	Nighttime Resting Respiratory Quotient (quiet Channel)	**ratio/30 min**
**24_QR_RQ_30**	/24 hr Resting Respiratory Quotient (quiet Channel)	**ratio/30 min**
**D_QA_EE_15**	Daytime Active Energy Expenditure (quiet Channel)	**(Kcal/15 min)**
**N_QA_EE_15**	Nighttime Active Energy Expenditure (quiet Channel)	**(Kcal/15 min)**
**24_QA_EE_15**	/24 hr Active Energy Expenditure (quiet Channel)	**(Kcal/15 min)**
**D_QA_RQ_15**	Daytime Active Respiratory Quotient (quiet Channel)	**ratio/15 min**
**N_QA_RQ_15**	Nighttime Active Respiratory Quotient (quiet Channel)	**ratio/15 min**
**24_QA_RQ_15**	/24 hr Active Respiratory Quotient (quiet Channel)	**ratio/15 min**
**Food/Day**	Food consumed/ day	**(g)**
**Food/Night**	Food consumed/ night	**(g)**
**Food/24**	/24 hr	**(g)**
**Water/Day**	Food consumed/ day	**(g)**
**Water/Night**	Food consumed/ night	**(g)**
**Water/24**	/24 hr	**(g)**
**BW**	Body Weight	**(g)**
**Ybreaks/Day**	Day Y beam breaks (activity)	#
**Ybreaks/Night**	Night Y beam breaks (activity)	#
**Ybreaks/24**	/24 hr Y beam breaks (activity)	#

**Xbreaks/Day**	Day X beam breaks (activity)	**#**
**Xbreaks/Night**	Night X beam breaks (activity)	**#**
**Xbreaks/24**	/24 hr X beam breaks (activity)	**#**
**Zbreaks/Day**	Day Rearings (activity/behavior)	**#**
**Zbreaks/Night**	Night Rearings (activity/behavior)	**#**
**Zbreaks/24**	/24 hr Rearings (activity/behavior)	**#**
**WheelMeters/Day**	Daytime Wheel meters run	**(m)**
**WheelMeterss/Night**	Nighttime Wheel meters run	**(m)**
**WheelMeterss/24**	/24 hr Wheel meters run	**(m)**
**WheelSpeed/Day**	Daytime wheel speed	**m/s**
**WheelSpeed/Night**	Nightime wheel speed	**m/s**
**WheelSpeed/24**	/24 hr wheel speed	**m/s**
**Run_pct/Day**	% of Day spent running	**(%)**
**Run_pct/Night**	% of Night spent running	**(%)**
**Run_pct/24**	% of 24 hr spent running	**(%)**
**PedMeters/Day**	Daytime in cage walking meters	**(m)**
**PedMeters/Night**	Nighttime in cage walking meters	**(m)**
**PedMeters/24**	/24 hr in cage walking meters	**(m)**
**PedSpeed/Day**	Daytime in cage walking speed	**m/s**
**PedSpeed/Night**	Nighttime in cage walking speed	**m/s**
**PedSpeed/24**	/24 hr in cage walking speed	**m/s**
**Walk_pct/Day**	% of Day spent walking	**(%)**
**Walk_pct/Night**	% of Night spent walking	**(%)**
**Walk_pct/24**	% of 24 hr spent walking	**(%)**
**Still_pct/Day**	% of Day spent staying still (<40sec)	**(%)**
**Still_pct/Night**	% of Night spent staying still (<40sec)	**(%)**
**Still_pct/24**	% of 24 hr spent staying still (<40sec)	**(%)**
**Sleep_pct/Day**	% of Day spent sleeping (>40sec)	**(%)**
**Sleep_pct/Night**	% of Night spent sleeping (>40sec)	**(%)**
**Sleep_pct/24**	% of 24 hr spent sleeping (>40sec)	**(%)**
**Sleep_hrs/Day**	Daytime Hours of Sleep (>40sec)	**(hrs)**
**Sleep_hrs/Night**	Nighttime Hours of Sleep (>40sec)	**(hrs)**
**Sleep_hrs/24**	/24 hr Hours of Sleep (>40sec)	**(hrs)**
**Total meters in cage**	Total meters run in cage for the length of run	**(m)**
**Total meters wheel**	Total meters runon wheel for the length of run	**(m)**
**Total Meters run**	Total meters run for the total run	**(m)**

**BW**	Body Weight	**(g)**
**aBMD**	Whole Body areal bone mineral Denisty	**g/cm2**
**aBMC**	Whole Body areal bone mineral Content	**(g)**
**Lean**	Lean muscle Mass	**(g)**
**Fat**	Fat Mass	**(g)**
**Total**	Total Mass	**(g)**
**% Fat**	fat mass/total mass	**%**
**F aBMD**	Femoral areal bone mineral Denisty	**g/cm2**
**F aBMC**	Femoral Body areal bone mineral Content	**(g)**

Genotype	Mouse ID	Sex	BW	D_Avg_EE	N_Avg_EE	24_Avg_EE
Pomc-cre/Dio3f/f	7632	M	45.9	0.434	0.515	0.474
Pomc-cre/Dio3f/f	7852	M	34.8	0.427	0.549	0.488
Pomc-cre/Dio3f/f	7974	M	35.1	0.438	0.627	0.532
Pomc-cre/Dio3f/f	7986	M	32.0	0.519	0.726	0.623
Pomc-cre/Dio3f/f	8039	M	27.9	0.451	0.693	0.572
Pomc-cre/Dio3f/f	2727	M	32.3	0.563	0.739	0.651
Pomc-cre/Dio3f/f	2730	M	29.9	0.568	0.756	0.662
Pomc-cre/Dio3f/f	3493	M	31.1	0.569	0.696	0.632
Pomc-cre/Dio3f/f	3495	M	40.8	0.594	0.669	0.631
Pomc-cre/Dio3f/f	3501	M	32.2	0.483	0.660	0.572
Pomc-cre/Dio3f/f	3505	M	46.4	0.585	0.613	0.599
Pomc-cre/Dio3f/f	3564	M	41.8	0.570	0.672	0.621
Pomc-cre/Dio3f/f	3565	M	33.1	0.557	0.734	0.645
Dio3f/f	7661	M	33.7	0.387	0.507	0.447
Dio3f/f	7665	M	37.9	0.406	0.603	0.504
Dio3f/f	7849	M	33.7	0.449	0.601	0.525
Dio3f/f	7853	M	31.7	0.505	0.648	0.577
Dio3f/f	7978	M	35.5	0.504	0.637	0.570
Dio3f/f	7979	M	30.8	0.421	0.589	0.505
Dio3f/f	7987	M	31.4	0.510	0.668	0.589
Dio3f/f	2729	M	36.1	0.603	0.804	0.704
Dio3f/f	2915	M	29.0	0.646	0.761	0.703
Dio3f/f	2916	M	36.1	0.562	0.740	0.651
Dio3f/f	2941	M	40.8	0.634	0.776	0.705
Dio3f/f	2943	M	35.4	0.527	0.669	0.598
Dio3f/f	3506	M	42.6	0.598	0.629	0.613
Dio3f/f	3563	M	42.9	0.543	0.626	0.585
	**Mean KO**		35.643	0.520	0.665	0.592
	**Mean control**		35.539	0.521	0.661	0.591
	**Student's t-test (P value)**		0.959	0.965	0.899	0.962

D_Avg_VO2	N_Avg_VO2	24_Avg_VO2	D_Avg_VCO2	N_Avg_VCO2	24_Avg_VCO2	D_Avg_RQ
1.49	1.75	1.620	1.233	1.511	1.372	0.827
1.50	1.92	1.709	1.100	1.422	1.261	0.733
1.52	2.17	1.845	1.164	1.731	1.447	0.761
1.78	2.48	2.128	1.481	2.123	1.802	0.826
1.56	2.37	1.968	1.219	1.985	1.602	0.776
2.00	2.60	2.30	1.37	1.87	1.62	0.685
2.01	2.64	2.33	1.40	1.97	1.68	0.693
1.96	2.38	2.17	1.59	2.01	1.80	0.802
2.06	2.31	2.19	1.60	1.84	1.72	0.770
1.67	2.26	1.97	1.32	1.89	1.61	0.784
2.04	2.10	2.07	1.55	1.74	1.64	0.758
2.00	2.34	2.17	1.46	1.80	1.63	0.729
1.95	2.54	2.25	1.44	2.00	1.72	0.736
1.34	1.75	1.544	1.054	1.413	1.233	0.782
1.41	2.08	1.743	1.095	1.682	1.389	0.775
1.57	2.10	1.838	1.322	1.564	1.361	0.733
1.77	2.25	2.007	1.317	1.763	1.542	0.744
1.32	2.19	1.977	1.787	1.787	1.552	0.744
1.47	2.04	1.756	1.608	1.608	1.354	0.745
1.42	2.26	2.011	1.418	1.989	1.704	0.803
2.15	2.86	2.51	1.41	1.93	1.67	0.653
2.30	2.71	2.50	1.41	1.81	1.67	0.665
1.41	2.60	2.29	1.90	1.90	1.65	0.709
2.26	2.76	2.51	1.87	1.87	1.69	0.666
1.29	2.36	2.11	1.29	1.69	1.49	0.686
2.09	2.17	2.13	1.55	1.73	1.64	0.739
1.89	2.17	2.03	1.46	1.72	1.59	0.773
1.811	2.297	2.054	1.379	1.837	1.608	0.760
1.692	2.307	2.068	1.464	1.747	1.538	0.730
0.335	0.931	0.894	0.321	0.200	0.255	0.100

N_Avg_RQ	24_Avg_RQ	D_QR_EE_30	N_QR_EE_30	24_QR_EE_30	D_QR_RQ_30	N_QR_RQ_30
0.861	0.844	0.441	0.462	0.451	0.833	0.857
0.733	0.733	0.410	0.411	0.410	0.723	0.708
0.795	0.778	0.419	0.477	0.448	0.743	0.773
0.856	0.841	0.485	0.508	0.496	0.799	0.821
0.835	0.806	0.488	0.427	0.457	0.757	0.804
0.722	0.704	0.511	0.517	0.514	0.639	0.765
0.741	0.717	0.527	0.494	0.510	0.639	0.742
0.839	0.820	0.539	0.493	0.516	0.781	0.801
0.791	0.781	0.514	0.538	0.526	0.751	0.763
0.834	0.809	0.568	0.599	0.584	0.838	0.856
0.822	0.790	0.566	0.521	0.544	0.742	0.814
0.767	0.748	0.595	0.572	0.584	0.727	0.749
0.788	0.762	0.569	0.495	0.532	0.756	0.762
0.808	0.795	0.338	0.381	0.359	0.775	0.806
0.806	0.790	0.379	0.388	0.383	0.752	0.750
0.741	0.737	0.402	0.401	0.401	0.704	0.714
0.780	0.762	0.433	0.438	0.436	0.730	0.715
0.813	0.778	0.481	0.502	0.492	0.751	0.774
0.786	0.766	0.421	0.430	0.426	0.738	0.763
0.876	0.840	0.492	0.506	0.499	0.802	0.861
0.675	0.664	0.581	0.548	0.564	0.634	0.679
0.668	0.667	0.632	0.586	0.609	0.639	0.662
0.731	0.720	0.556	0.531	0.544	0.711	0.761
0.679	0.673	0.610	0.597	0.603	0.666	0.673
0.716	0.701	0.489	0.481	0.485	0.690	0.704
0.795	0.767	0.546	0.542	0.544	0.717	0.741
0.793	0.783	0.523	0.535	0.529	0.804	0.775
0.799	0.780	0.510	0.501	0.506	0.748	0.786
0.762	0.746	0.492	0.490	0.491	0.722	0.741
0.094	0.093	0.534	0.667	0.584	0.250	0.029

24_QR_RQ_30	D_QA_EE_15	N_QA_EE_15	24_QA_EE_15	D_QA_RQ_15	N_QA_RQ_15	24_QA_RQ_15
0.845	0.573	0.658	0.615	0.845	0.862	0.853
0.716	0.587	0.642	0.615	0.757	0.767	0.762
0.758	0.633	0.719	0.676	0.780	0.797	0.788
0.810	0.687	0.774	0.730	0.844	0.832	0.838
0.780	0.630	0.767	0.699	0.792	0.844	0.818
0.702	0.803	0.832	0.818	0.694	0.714	0.704
0.691	0.773	0.895	0.834	0.694	0.753	0.723
0.791	0.831	0.867	0.849	0.870	0.887	0.879
0.757	0.784	0.717	0.751	0.810	0.800	0.805
0.847	0.625	0.718	0.672	0.827	0.833	0.830
0.778	0.729	0.781	0.755	0.830	0.851	0.840
0.738	0.756	0.791	0.774	0.777	0.776	0.777
0.759	0.735	0.821	0.778	0.769	0.778	0.774
0.791	0.559	0.563	0.561	0.812	0.778	0.795
0.751	0.527	0.646	0.586	0.791	0.805	0.798
0.709	0.532	0.763	0.648	0.743	0.756	0.749
0.722	0.696	0.696	0.696	0.783	0.770	0.776
0.762	0.678	0.712	0.695	0.780	0.799	0.789
0.750	0.565	0.624	0.595	0.800	0.787	0.793
0.832	0.603	0.737	0.670	0.815	0.879	0.847
0.656	0.794	0.897	0.845	0.661	0.666	0.663
0.650	0.805	0.865	0.835	0.708	0.675	0.691
0.736	0.762	0.813	0.787	0.749	0.742	0.745
0.669	0.800	0.829	0.814	0.685	0.683	0.684
0.697	0.719	0.758	0.738	0.737	0.710	0.723
0.729	0.735	0.740	0.738	0.779	0.829	0.804
0.789	0.674	0.705	0.689	0.823	0.808	0.815
0.767	0.703	0.768	0.736	0.792	0.807	0.799
0.732	0.675	0.739	0.707	0.762	0.763	0.762
0.085	0.438	0.391	0.388	0.151	0.054	0.081

Food/Day	Food/Night	Food/24	Water/Day	Water/Night	Water/24
1.047	2.712	3.758	0.824	1.607	2.431
0.805	1.083	1.888	0.303	1.005	1.308
0.884	1.896	2.780	0.716	2.214	2.930
2.367	3.755	6.122	1.597	3.910	5.507
1.071	3.542	4.613	0.502	2.470	2.972
1.06	2.30	3.36	0.92	2.25	3.17
1.33	2.71	4.04	0.91	2.06	2.97
2.00	3.61	5.61	1.98	3.63	5.61
0.78	1.81	2.59	1.28	1.73	3.01
1.99	3.08	5.08	1.78	2.41	4.19
1.01	2.06	3.07	0.55	1.25	1.80
0.64	1.70	2.33	0.51	1.65	2.16
0.59	1.99	2.57	0.28	1.57	1.85
0.459	0.801	1.260	1.205	1.402	2.606
0.995	3.223	4.218	0.675	2.341	3.016
0.526	0.821	1.347	0.871	1.197	2.068
0.624	1.929	2.553	0.652	1.817	2.469
0.968	3.859	4.827	0.772	2.609	3.381
0.388	1.762	2.150	0.523	2.245	2.768
0.516	1.896	2.413	0.927	4.351	5.278
0.62	1.31	1.93	0.81	1.23	2.04
1.11	1.49	2.60	0.64	1.05	1.69
0.51	1.20	1.72	0.46	1.91	2.37
0.48	1.51	1.99	0.22	1.24	1.46
0.64	1.56	2.20	0.40	1.64	2.05
1.05	2.18	3.22	0.92	1.43	2.35
0.86	2.24	3.09	0.47	1.49	1.96
1.198	2.481	3.679	0.935	2.136	3.070
0.695	1.842	2.538	0.682	1.854	2.536
0.006	0.060	0.019	0.140	0.399	0.236

Ybreaks/Day	Ybreaks/Night	Ybreaks/24	Xbreaks/Day	Xbreaks/Night	Xbreaks/24	Zbreaks/Day	Zbreaks/Night
10084	39167	49250	5833	9921	15754	2431	12728
4916	7903	12819	5816	8572	14387	1176	2514
6600	13059	19658	6005	12418	18424	1435	4087
11049	15275	26324	9204	13643	22847	2999	4890
5476	14146	19622	23201	12817	36018	1065	3758
9610	11447	21057	8155	11422	19577	1556	3885
9981	11963	21944	9247	11485	20731	2095	3703
8589	14716	23305	8769	15567	24336	4328	6693
9463	9458	18920	5899	8958	14857	3158	4800
7779	13413	21192	7560	14931	22491	3848	7980
5641	6774	12415	6383	8225	14608	3391	4302
8983	12713	21695	9077	13434	22511	1900	5004
5464	9776	15239	362	0	362	1620	7141
11232	13388	24620	9658	11977	21635	1733	3185
9054	14995	24049	7071	13405	20476	1638	4487
9281	10146	19427	8969	9423	18392	1784	2300
7047	11395	18442	5637	9977	15614	1393	3810
7693	10711	18404	7295	9236	16531	2101	7533
7117	10873	17990	7060	11262	18322	1953	3368
7397	10580	17977	6194	9339	15532	1740	5049
9331	7721	17051	10306	8189	18495	1959	2157
9718	12244	21962	9193	11505	20698	3532	4858
10727	12902	23629	8043	11318	19361	2499	4210
9753	13707	23461	8328	11883	20210	3108	5715
9032	13296	22329	2707	3341	6048	2719	5951
10117	12163	22279	9584	13583	23167	4017	5862
9816	15042	24857	9720	14313	24033	2529	4780
7971.667	13831.333	21803.000	8116.141	10876.308	18992.449	2384.718	5498.590
9093.750	12082.964	21176.857	7840.321	10625.024	18465.274	2336.024	4518.833
0.108	0.438	0.809	0.854	0.851	0.833	0.892	0.245

Zbreaks/24	WheelMeters/Day	WheelMeterss/Night	WheelMeterss/24	WheelSpeed/Day	WheelSpeed/Night
15159	131	904	1035	0.166	0.180
3690	91	2311	2402	0.137	0.232
5522	241	4016	4257	0.175	0.273
7889	484	3411	3895	0.171	0.199
4823	104	6241	6345	0.138	0.283
5441	554	5222	5776	0.193	0.245
5797	291	5443	5734	0.187	0.287
11021	406.9	1782.9	2189.8	0.155	0.180
7958	732.9	2985.3	3718.2	0.164	0.195
11828	300.9	3212.5	3513.4	0.184	0.228
7693	158.7	453.1	611.9	0.139	0.154
6904	83.1	2358.1	2441.2	0.167	0.206
8761	23.5	4753.6	4777.1	0.107	0.251
4918	223	2213	2436	0.173	0.219
6125	168	4066	4234	0.163	0.237
4084	1081	4794	5875	0.180	0.242
5203	138	4426	4564	0.177	0.266
9634	9	2065	2073	0.105	0.230
5321	2217	4996	7213	0.202	0.238
6789	288	2497	2784	0.164	0.219
4116	461	7431	7891	0.185	0.271
8390	298	3435	3733	0.166	0.205
6709	190	4563	4753	0.146	0.223
8823	217	3765	3983	0.154	0.208
8669	205	3624	3829	0.159	0.210
9879	210.6	803.3	1013.9	0.144	0.165
7309	266.6	1813.0	2079.6	0.140	0.178
7883.308	277.096	3314.876	3591.972	0.160	0.224
6854.786	426.510	3606.492	4033.073	0.161	0.222
0.321	0.385	0.664	0.550	0.919	0.905

WheelSpeed/24	Run_pct/Day	Run_pct/Night	Run_pct/24	PedMeters/Day	PedMeters/Night	PedMeters/24
0.173	2.238	13.046	7.642	23.140	37.058	60.199
0.185	1.545	23.915	12.730	25.704	37.428	63.132
0.224	3.704	36.539	20.121	33.870	98.587	132.457
0.185	6.565	41.571	24.068	35.234	77.424	112.658
0.211	1.543	53.877	27.710	38.828	149.623	188.451
0.219	7.2	51.1	29.1	32	57	89
0.237	3.9	45.7	24.8	30	55	85
0.168	6.9	24.5	15.7	60.1	110.9	171.0
0.180	10.7	35.5	23.1	96.3	125.5	221.7
0.206	4.0	33.9	19.0	41.9	99.2	141.1
0.147	3.2	7.8	5.5	42.1	51.4	93.5
0.187	1.2	27.3	14.3	43.5	75.1	118.6
0.179	0.5	45.7	23.1	41.6	81.4	123.0
0.196	3.359	23.681	13.520	40.580	68.515	109.096
0.200	2.724	41.501	22.113	32.102	87.672	119.773
0.211	14.604	47.011	30.808	40.433	52.702	93.136
0.222	2.057	40.832	21.444	29.059	85.188	114.247
0.168	0.222	21.687	10.954	43.493	68.082	111.575
0.220	25.593	49.372	37.482	30.165	71.466	101.631
0.191	4.241	27.100	15.671	35.925	74.228	110.153
0.228	6.2	65.0	35.6	57	64	121
0.185	5.0	40.9	22.9	53	69	122
0.185	3.0	48.4	25.7	42	77	119
0.181	3.6	43.2	23.4	54	73	127
0.185	3.3	42.1	22.7	59	102	161
0.155	4.4	13.4	8.9	50.3	60.8	111.2
0.159	4.2	24.1	14.1	26.5	61.5	88.0
0.192	4.090	33.884	18.987	41.883	81.215	123.099
0.192	5.890	37.732	21.807	42.366	72.521	114.888
0.972	0.373	0.482	0.375	0.935	0.385	0.555

PedSpeed/Day	PedSpeed/Night	PedSpeed/24	Walk_pct/Day	Walk_pct/Night	Walk_pct/24	Still_pct/Day
0.010	0.011	0.010	11.749	12.077	11.913	88.014
0.010	0.009	0.010	8.244	11.289	9.767	92.686
0.013	0.014	0.013	9.067	17.593	13.330	89.316
0.011	0.013	0.012	11.459	17.039	14.249	79.154
0.013	0.015	0.014	10.603	19.123	14.863	89.585
0.012	0.012	0.012	10.1	30.5	11.9	88.6
0.012	0.013	0.013	9.5	12.8	11.1	92.1
0.013	0.014	0.014	16.1	24.3	20.2	85.4
0.012	0.012	0.012	26.2	30.2	28.2	78.1
0.013	0.014	0.014	12.8	40.7	16.6	89.9
0.011	0.010	0.010	14.5	18.1	16.3	90.7
0.011	0.012	0.011	17.5	21.3	19.4	91.6
0.011	0.010	0.010	18.7	21.2	20.0	93.3
0.012	0.014	0.013	13.524	15.753	14.639	86.225
0.013	0.014	0.013	9.501	18.099	13.800	88.640
0.013	0.013	0.013	10.864	12.657	11.761	75.524
0.011	0.012	0.012	9.787	17.211	13.499	89.082
0.009	0.011	0.010	15.282	18.870	17.076	88.865
0.014	0.015	0.014	6.754	14.161	10.457	66.545
0.012	0.013	0.013	9.892	16.957	13.424	86.058
0.015	0.014	0.015	12.4	9.0	10.7	87.8
0.012	0.013	0.013	16.5	14.8	15.6	88.0
0.014	0.014	0.014	11.7	15.5	13.6	91.9
0.014	0.012	0.013	14.1	17.6	15.9	89.5
0.011	0.011	0.011	20.0	21.8	20.9	88.7
0.012	0.013	0.013	15.6	17.4	16.5	89.5
0.011	0.012	0.012	9.4	47.9	13.3	92.3
0.012	0.012	0.012	13.578	21.242	15.982	88.344
0.012	0.013	0.013	12.518	18.408	14.366	86.333
0.103	0.230	0.112	0.529	0.408	0.304	0.393

Still_pct/Night	Still_pct/24	Sleep_pct/Day	Sleep_pct/Night	Sleep_pct/24	Sleep_hrs/Day	Sleep_hrs/Night
71.176	79.595	82.997	66.141	74.569	9.960	7.937
67.127	79.906	89.753	62.153	75.953	10.771	7.458
46.030	67.673	85.703	39.603	62.653	10.285	4.752
39.525	59.339	74.601	32.278	53.439	8.952	3.873
24.642	57.113	85.191	18.227	51.709	10.223	2.187
43.6	66.1	83.9	34.2	59.1	10.1	4.1
49.3	70.7	87.2	41.2	64.2	10.5	4.9
63.8	74.6	76.3	49.5	62.9	9.2	5.9
51.1	64.6	63.6	32.7	48.2	7.6	3.9
56.5	73.2	83.2	45.3	64.3	10.0	5.4
85.2	87.9	82.4	74.1	78.3	9.9	8.9
63.9	77.7	82.0	50.5	66.2	9.8	6.1
46.9	70.1	83.5	31.5	57.5	10.0	3.8
63.475	74.850	80.601	56.252	68.426	9.672	6.750
41.421	65.031	85.773	34.258	60.016	10.293	4.111
43.371	59.448	70.889	36.975	53.932	8.507	4.437
40.777	64.929	84.540	32.812	58.676	10.145	3.937
55.191	72.028	82.556	48.428	65.492	9.907	5.811
34.035	50.290	62.655	26.886	44.770	7.519	3.226
53.469	69.763	82.983	46.734	64.858	9.958	5.608
31.4	59.6	81.1	25.2	53.2	9.7	3.0
52.9	70.5	78.5	43.7	61.1	9.4	5.2
45.1	68.5	85.5	35.1	60.3	10.3	4.2
49.0	69.2	81.9	38.1	60.0	9.8	4.6
49.5	69.1	78.1	35.2	56.7	9.4	4.2
80.2	84.9	79.4	69.7	74.5	9.5	8.4
68.9	80.6	86.1	57.5	71.8	10.3	6.9
54.512	71.428	81.569	44.416	62.993	9.789	5.330
50.631	68.482	80.042	41.918	60.984	9.607	5.026
0.495	0.388	0.551	0.653	0.547	0.554	0.649

Sleep_hrs/24	aBMD	aBMC	Lean	Fat	total	%Fat	FaBMD	FaBMC
17.897	0.0536	0.536	27.8	16.2	44.1	36.8	0.0683	0.011
18.229	0.05	0.47	23.2	9.4	32.6	28.8	0.0662	0.01
15.037	0.0507	0.471	23.2	7.5	30.7	24.4	0.0694	0.011
12.825	0.0557	0.551	26.8	3.3	30.1	10.9	0.0809	0.013
12.410	0.0509	0.449	21.5	4.1	25.6	16.2	0.0653	0.011
14.2	0.0537	0.557	25.9	7.1	33.1	21.5	0.0786	0.011
15.4	0.057	0.565	24.5	5.4	29.9	18.2	0.0777	0.011
15.1	0.0528	0.527	24.1	3.8	27.9	13.5	0.0767	0.011
11.6	0.0528	0.547	25.4	11.9	37.3	31.8	0.0763	0.011
15.4	0.055	0.581	25.3	4.1	29.4	14.0	0.0756	0.011
18.8	0.0499	0.497	24.3	19.5	43.8	44.6	0.0661	0.008
15.9	0.0523	0.445	24.5	14.7	39.2	37.5	0.0631	0.008
13.8	0.0507	0.458	22.8	8.6	31.4	27.4	0.0628	0.008
16.422	0.0512	0.453	24.9	6.9	31.8	21.7	0.0609	0.01
14.404	0.0512	0.486	23	12.6	35.6	35.5	0.0593	0.01
12.944	0.0508	0.441	22.3	9.2	31.5	29.3	0.062	0.01
14.082	0.0504	0.43	22.2	6.8	28.9	23.4	0.0625	0.01
15.718	0.0499	0.449	22.8	10.1	32.9	30.6	0.0607	0.01
10.745	0.049	0.481	20	8.5	28.4	29.8	0.0659	0.01
15.566	0.0516	0.489	25.6	2.8	28.4	9.9	0.0763	0.012
12.8	0.0542	0.481	22.7	12.7	35.3	35.8	0.0669	0.009
14.7	0.0559	0.581	27	13.1	40	32.7	0.0798	0.011
14.5	0.0537	0.524	25.6	11.2	36.8	30.5	0.0718	0.01
14.4	0.0531	0.528	26.2	18	44.2	40.8	0.0787	0.011
13.6	0.0507	0.496	22.1	13.9	36	38.6	0.0645	0.009
17.9	0.049	0.524	19.7	20.2	39.9	50.6	0.0587	0.008
17.2	0.0533	0.452	24.8	16	40.8	39.3	0.0649	0.008
15.118	0.053	0.512	24.562	8.892	33.469	25.046	0.071	0.010
14.640	0.052	0.487	23.493	11.571	35.036	32.036	0.067	0.010
0.550	0.245	0.161	0.178	0.173	0.461	0.085	0.089	0.305

Supplementary Table S2

Gene symbol	Forward primer 5'–3'	Reverse primer 5'–3'
Adipq	GGAATGACAGGAGCTGAAGGGC	ACAGTGACGCGGGTCTCCAGC
Agrp	GCAGACCGAGCAGAAGAAGT	ATTGAAGAAGCGGCAGTAGC
Bcar3	GTCGCACAGATGCCTATCAAG	CACAGGACCGTATGGAAGGAG
Bmpr1a	GTGTGTGAAACGCTTGCGGCCAATC	GTGAGTCTGGAGGCTGGATTATGGG
Crh	CCTGAGAGAAGTCCCTCTG	ATCAGAACCGGCTGAGGTTG
Dio2	CCTCCTAGATGCCTACAAACAGG	CATTCGGCCCCATCAGCGGTC
Dusp1	ACAGCTTCCTGTCCATCCTG	CTGAGAGAGGCTCATGGACG
Elovl3	GGAAGAGCTTCAGCTTGCAG	GCTTGAGGCCCACTGTAAAC
Fos	CGGCATCATCTAGGCCCAG	TCTGCTGCATAGAAGGAACCG
Homer1	TTGAGTTGCCTCCGGAAAAGA	TTGTGTTCGGGTCAATCTGGA
Hr	AAC CCT GCA TCC AAG TAG CA	AGC ACT GTG TGG CAT GTG TT
Irf4	TGTGGGAGAACGAGGAGAAGAGCG	GTCGATCCCTTCTCGGAACTTGCCT
Klf9	GGCTGTGGGAAAGTCTATGG	AAGGGCCGTTCACCTGTATG
Lep	GGAGACCCCTGTGTCGGTTCCT	GCGGATACCGACTGCGTGTGTG
Lepr	GTGTACACCTCTGAAGAAAGATGATGTGTC	CCAGGGAGAGATTGGATATGCCAGGT
Lpl	CCTTCGTGGTGATCCATGGATGG	GGGCCCGATACAACCAGTCTACT
Malat1	CTTTGCGGGTGTTGTAGGTTT	GAGGCTTGTGGTAGGTCATCTGTT
Mc4r	CCGGACGGAGGATGCTATGAGCA	GGCTATCGCCACGATCACTAGAATG
Med1	GGTGATGGATGTGCAAGACTCAACACATG	CCTCCGAATAGCCCTCATCGTCACA
Ncoa2	GAGCAGCATGAACCAGATGACAGGC	GGCAGTTGGTTTGGGAAAAGGTTGCC
Nirp1	GCGTGGACTGTGAGCGGTTGCA	GTGGGAAAGGAAGCTGAGAAGGCTG
Npy	TACTACTCCGCTCTGCGACA	TCACCACATGGAAGGGTCTT
Nr1h3	GTCCTTGTGGCTGGAGGCCTCA	CTCTGCTGACTCCAACCCTATCCCT
Pcsk1	CCATCCTCGGAGGTCCCGAAGA	GGGCAGAGCTGCAGTCATTCTGG
Pcsk2	CCCCTTTGCAGAAGGCCTGTATCAC	CAAATCCTTCTTGTTGCAGCGCCATCT
Pgc1a	GTGAGGACCAGCCTCTTTGCCC	GTCGCTACACCACTTCAATCCACC
Pomc	CACCACGGAGAGCAACCT	GTTTTCAGTCAGGGGCTGTT
Pparg	CTCCATAAAGTCACCAAAGGGCTTCC	CTACACGATGCTGGCCTCCCTG
Prdm16	TGCTGACGGATACAGAGGTGT	CCACGCAGAACTTCTCGCTAC
Scd1	TTCTTGCGATACACTCTGGTGC	CGGGATTGAATGTTCTTGTCGT
Trh	GGCTCAGCATCTTGGAAAGCTCTGC	CAAGGCGCAGGATTTGGGGATACCA
Tshb	CAACACCACCATCTGTGCTG	CAGACATCCTGAGAGAGTGC
Ucp1	CTCACTCAGGATTGGCCTCTACGA	GAACACTGCCACACCTCCAGTC
Ucp2	CGAGGGGATCGGGCCATGGTA	GATTTCCTGCTACCTCCCAGAAGATGG
